# Paediatric Preparedness: Document Analysis of the Challenges Experienced Using Smartwatch Technologies to Support Children Living with a Chronic Health Condition

**DOI:** 10.3390/ijerph22020133

**Published:** 2025-01-21

**Authors:** Sonia Butler, Dean Sculley, Derek Santos, Xavier Gironès, Davinder Singh-Grewal, Andrea Coda

**Affiliations:** 1 School of Bioscience and Pharmacy, University of Newcastle, Ourimbah, NSW 2258, Australia; dean.sculley@newcastle.edu.au; 2 School of Health Sciences, Queen Margaret University, Edinburgh EH21 6UU, UK; dsantos@qmu.ac.uk; 3 Department of Research, Universities de Catalunya, Generalitat de Catalunya, 08003 Barcelona, Spain; xaviergirones@gencat.cat; 4 Department of Rheumatology, Sydney Children’s Hospitals Network (Randwick), Randwick, NSW 2031, Australia; davinder.singhgrewal@health.nsw.gov.au; 5 Department of Rheumatology, Sydney Children’s Hospitals Network (Westmead), Westmead, NSW 2145, Australia; 6 John Hunter Children’s Hospital, New Lambton Heights, NSW 2305, Australia; 7 Discipline of Child and Adolescent Health, University of Sydney, Camperdown, NSW 2050, Australia; 8 School of Women’s and Children’s Health, University of NSW, Sydney, NSW 2052, Australia; 9 School of Health Sciences, University of Newcastle, Callaghan, NSW 2308, Australia; andrea.coda@newcastle.edu.au; 10 Equity in Health and Wellbeing Research Program, The Hunter Medical Research Institute (HMRI), Newcastle, NSW 2305, Australia

**Keywords:** app, chronic health condition, digital health, integrated care, paediatric, smartwatch, wearable technology

## Abstract

Smartwatch technology is increasingly being used to support the management of chronic health conditions. Yet, many new digital health innovations fail because the correct foundations are not well established. This exploratory study aims to uncover the challenges experienced during the setup phase of a smartwatch intervention, to support the prototype development of a digital health intervention for children. Five children with a chronic health condition were asked to wear a smartwatch for 14 days that collects health data (pain levels, medication adherence, and physical activity performance). To explore the experiences of these children, their parents and the research team, all written records were analysed using READ’s four steps of document analysis and reported using the Standards for Reporting Qualitative Research checklist. The following three themes emerged: 1.) Infrastructure limitations: inexpensive smartphones prevented connection, and outpatient clinics’ internet black spots constrained setup and training; 2.) Personal phone restrictions: limited setup, training, and engagement; 3.) Elimination of the parent’s phone: provided children with digital support (a smartphone, pre-installed apps, cellular data) to allow active participation. Overall, we identified barriers hindering the use of smartwatch technology in clinical practice. More resources are needed to ensure paediatric preparedness for digital health support.

## 1. Introduction

Over the last fifty years, the number of children living with a chronic health condition has steadily increased. This has been primarily due to advancements in public health, improving nutrition, and the medical management of childhood infections and neonatal complications, which have decreased childhood mortality and improved the survival of many children with a chronic health condition [1,2]. Typically, the definition of a chronic health condition entails functional limitations due to a health condition, making the child dependent on healthcare interventions, such as medications, assistive devices, or medical care [3]. This covers a wide range of disorders, including special healthcare needs, medical complexities, and chronic disease [4]. A roughly estimation of their prevalence across the US, Europe, and Australia ranges between 8.4 and 45% of all children [5,6,7], and half of these children are also in need of comprehensive healthcare services [7], spanning across their lifetime [4]. This is rapidly bringing about new challenges and costs for healthcare services [8], making the clinical management of chronic health conditions in childhood a global health challenge [5,6,7].

Understandably, providing children with a chronic health condition with the correct support is complex. Their support needs constantly change according to their age and the stage of their developmental trajectory [9]. Generally, this support involves an interdisciplinary healthcare team that works collaboratively with the child and their family [10]. The main goals of care are reducing ongoing illness and risk of hospitalisation [11], retaining functional ability [12], and avoiding permanent disability and/or premature death [11,13]. Care must also address the psychological and financial pressures placed on a child and their family, straining their personal resilience [14], social relationships [12], economic resources [15], and overall life satisfaction [6]. Therefore, to minimise these negative effects, it is essential that interventions are delivered early to optimise physical, mental, and emotional wellbeing [16]. It is also important to ensure that this support is ongoing to reduce the impact throughout childhood and the school years [12], to ensure a good quality of life [17]. In addition, this support needs to promote the development of the child and their family’s self-management skills to improve health outcomes and their ability to deal with complications when they arise [9].

For healthcare systems to adapt and respond to the increasing needs of children with chronic health conditions, there is a need for sustained investment to find cost-effective innovative solutions [4,8,16]. In Australia, this need has been highlighted in the National Action Plan for the Health of Children and Young People 2020–2030, which emphasises the importance of seeking new approaches to improve health promotion and enhance support [16], changing the focus of healthcare from a disease-specific approach to a more preventative and self-managing approach [18]. The utilisation of digital technology has been gaining attention in recent years, and has the potential to improve disease management and support integrated care [19,20,21].

Major advancements and the widespread use of digital technology have now created an international landscape that could be utilised to shift the focus of healthcare delivery towards a patient-centred model [22,23,24], with many digital devices, particularly wearables, being placed at the forefront of patient care to support self-management skill development and improve communication between the child, parents, and healthcare team [25,26]. For children, smartwatch studies have focused on using this technology for education [27], bedtime routines [28], and self-regulatory behaviour [29]. Also, the inbuilt sensors within the watch have been used to record a wide range of physiological, behavioural, and environmental health data, for example, on arrhythmias [30], heart rate, skin temperature, scratching, seizures, light exposure, and physical activity levels [25], thus automating the collection of health data. This data can then be transferred to a smartphone to be consolidated and stored, and reviewed by the user or sent to a healthcare provider [31] or web-based health platform and utilised by the entire interdisciplinary team [26,32], allowing for their inputs and evaluations to provide more holistic management and an improved treatment environment. This suggests that this corpus of data could be used as a decision-making tool to improve the quality of life of children living with a chronic health condition [31,32].

Yet, most digital health studies published to date are at a very early stage of development [25,27,30,33]. Also, many new digital health innovations fail because the right foundations were not put into place before they were started [24], and the contextual environment was not examined to determine if it could absorb and sustain the intervention [34]. Therefore, it is important to identify the central tenets needed to implement and receive digital healthcare using smartwatch technology to ensure the practicality of smartwatch use within the healthcare setting, particularly before conducting any costly trials [24,35].

The main purpose of this study is to explore the challenges experienced during the setup phase of a smartwatch intervention designed to support children living with a chronic health condition.

## 2. Materials and Methods

This study was guided by the Standards for Reporting Qualitative Research Checklist [36] to ensure comprehensive reporting and transparent results.

### 2.1. Qualitative Approach and Research Paradigm

This study employed the qualitative research method called document analysis to better understand the prerequisite requirements needed to support Australian children using smartwatch technology. Qualitative research is commonly used to identify problems and facilitate improvements by those intended to benefit from the intervention [37]. In addition, document analysis supports the use of documents as the data source [37,38]. It also provides a systematic approach to assist with the examination of a large amount of written data, particularly for the interpretation of past events that can no longer be observed or may have been forgotten [38].

### 2.2. Researcher Characteristics and Reflexivity

Document analysis was conducted by three research team members (S.B., D.S. [Dean Sculley], A.C.). S.B. is an experienced qualitative researcher. S.B. was directly involved in the recruitment and setup, and therefore participants felt comfortable in providing their feedback. D.S. [Dean Sculley] and A.C. are doctoral supervisors. D.S. [Dean Sculley] holds a PhD in Human Biology, and A.C. holds one in Health Science. D.S. [Dean Sculley] and A.C. were not actively involved with the participants and therefore offered more objective views and further insight. All other manuscript authors (D.S. [Derek Santos], X.G., D.S.-G.) reviewed the final themes.

### 2.3. Context

InteractiveClinics is a chronic disease web-based platform, developed by researchers at the University of Newcastle, Australia and the University of Manresa, Spain to support digital innovation. The central components are a commercially available smartwatch and customised phone app that collects health data, which can be viewed by the child in the app and by the child, parents, and research team on the web-based platform to support family-centred integrated healthcare.

For this exploratory study, the health data collected focused on several key areas that can impair health outcomes for children living with a chronic health condition: symptoms and treatment monitoring [7] and self-management skill development [9]. This included daily pain monitoring, medication usage, and physical activity (low-intensity and moderate-to-vigorous intensity) levels [7,9,20].

Participation in this study involved children wearing a smartwatch for two weeks. The main purpose of wearing the smartwatch throughout the day was to promote consistent monitoring and engagement with the intervention to support data collection.

To promote user engagement, automated notifications were sent daily to the smartwatch at a time predetermined by the child/parent during setup. These notifications aimed to remind children to record their pain levels, prompt medication adherence, and record medication usage (including the need to take PRN medication) in the app. An automatic feedback loop was embedded into the system, so the child received immediate feedback on their data entry to help them stay motivated. Additionally, the smartwatch triggered notifications throughout the day to encourage increased physical activity and automatically transferred the child’s physical activity levels to the app and web-based platform, where daily and weekly graphs were generated for the child and their parents to review. The child, parents, and research team could also review these results on the web-based platform.

### 2.4. Materials

All children were provided with a TicWatch GTH smartwatch, which is commercially available through Mobvoi. The watch was selected because of the price (AUD $100), water rating (up to 50 metres), and extended battery life (10 days) [39].

Also, at this very early stage of the intervention’s prototype development, all children/parents were required to download three free apps from the Google Play Store onto their smartphone (supported by an Android operating system [OS]). 

These apps included: the Mobvoi app (RH239EV000798_17212, ShenzhenUltraEasy Technology Co., Ltd., Shenzhen, China), Google Fit Activity Tracking (Wear OS) (2.84.2, Mountain View, CA, USA), and InteractiveClinics app (University of Newcastle, Manresa, Callaghan, Australia; University of Manresa, Spain). This would allow the transference of data from the commercially available watch to the InteractiveClinics phone app and the web-based platform.

All children and parents were also supplied with support and training (15 min), and a prepaid envelope to return the watch to the research team at the end of the study.

### 2.5. Sample Strategy

Using purposive sampling, five children were recruited from a subspecialty outpatients clinic within a specialised tertiary referral paediatric hospital in a regional city in NSW, Australia, between May and June 2022. Children were eligible to participate in the study if they were aged between 10 and 18 years and had access to a smartphone with an Android operating system (OS). Children were excluded if they owned a smart watch or had a cognitive impairment or physical disability that would affect their ability to use digital technology.

### 2.6. Ethics

Ethics approval for this study was granted by the Research Ethics Committee (ref no: 2019/ETH01035). All children and their parent/s received an information sheet explaining the study and provided signed informed consent. To ensure anonymity, all identifying details were removed and replaced with a participant number. Participants were also informed that they could withdraw from the study at any time, without prejudice, by simply returning the watch in the supplied prepaid envelope to the research team.

### 2.7. Data Collection

Documentation began during the setup phase of the intervention and concluded at the end of the study (14 days). This included field notes after setup and training (S.B.), phone call memos (parents, S.B., A.C.), text messages (children, parents, S.B., A.C.), emails (parents, S.B., D.S. [Dean Sculley], A.C.),responses (parents, S.B., A.C.), and research team meeting minutes (S.B., D.S. [Dean Sculley], A.C.). All documentation aimed to do the following: 1.) provide a clear picture of how the intervention was performing; 2.) record problems and challenges that children, parents and the research team experienced; 3.) record decisions that supported changes in the intervention’s development. This allowed for the intervention’s progress to be tracked over a period of time [38].

Additionally, feedback was provided weekly from S.B. to A.C., and a list of what was working and what needed improving was then forwarded to A.C. and D.S. [Dean Sculley]. This enhanced the team meeting discussions and further documentation (S.B., D.S. [Dean Sculley], A.C.).

### 2.8. Data Analysis

Using READ’s four steps of document analysis, a step-by-step guide was provided for data analysis [40]. These four steps were ready materials, extract data, analyse data, and distil approach.

(1) Ready materials: This first step involved identifying all the documents that would be used in this study. These documents included field notes, phone call memoranda, emails and text messages, and research team meeting minutes.

(2) Extract data: This second step entailed extracting all the data related to the aim of this study. Initially, all the documents needed to be skimmed over to obtain an overview and then re-read to identify specific categories [40]. This process allowed the data to be organised in a timeline of events within an excel spreadsheet by S.B. Then, all data excerpts were meticulously checked and rechecked to ensure that they retained their original meaning.

(3) Analyse data: This third step began once all the data were extracted. This ensures that a full picture was gained and a comprehensive understanding of the research aim was being developed [38,40]. This step allowed for the challenges experienced during the setup phase of this study to be identified (by S.B., A.C., and D.S. [Dean Sculley]) and initial codes to be formed.

(4) Distil approach: In this final step, codes were further refined, illustrated with quotes, and grouped into thematic categories. All themes were internally reviewed (by S.B., D.S. [Dean Sculley], D.S. [Derek Santos], X.G., D.S.-G., and A.C.) to ensure confidence in the results. The challenges experienced during the setup phase of the smartwatch intervention were clearly understood and no new challenges emerged from the data, allowing data saturation to be reached.

## 3. Results

**Figure 1:**
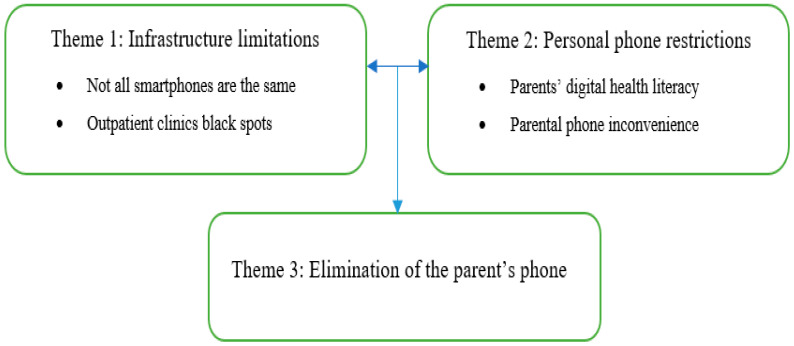
Main themes.

Five children aged 11–17 years (mean 13.6, SD 2.7, female 60%, three-fifths) and their parents (n = 5) participated in the study. All children were living with the chronic health condition, juvenile idiopathic arthritis. Analysis of all the written documentation collected from children, parents, and the research team (field notes, phone call memoranda, emails and text messages, and research team meeting minutes) resulted in the formation of three main themes to support the development of digital technology in paediatrics. These included infrastructure limitations, personal phone restraints, and the elimination of the parents’ phone (Figure 1).

### 3.1. Theme 1: Infrastructure Limitations

During the setup phase of this study, it soon became clear to the research team that several infrastructure limitations were hindering the delivery of the intervention to children. This resulted in the following subthemes: not all smartphones are the same, and outpatient clinics black spots.

#### 3.1.1. Not All Smartphones Are the Same

Not all smartphones using an Android operating system (OS) were the same. Parents’ phones varied in price from AUD $100 to AUD $800. We soon realised that the price of the phone reflected the quality of the processor/s (CPU) and RAM, influencing the phone’s performance and connectivity to the smartwatch. This added an additional layer to recruitment. Parents needed to check if their phone was compatible with the necessary apps by viewing them in the App Store. If their phone was not compatible, the following message would be seen, “Your device is not compatible with this version”, indicating that they could not use the intervention.

#### 3.1.2. Outpatients Clinic Black Spots

A significant challenge impacting on the setup of the intervention was gaining internet access in the outpatients clinic, despite the hospital offering free Wi-Fi services. The positioning of the outpatients clinic, combined with long corridors and small consultation rooms, placed the clinic in an internet black spot. For this study, this meant that all smartphones had either no signal or a weak signal that was too unstable to maintain connectivity to download the required apps needed for the intervention. This resulted in no participants (0%, 0/5) being able to connect the smartphones to the smartwatches in the clinic.

Instead, potential participants, with a research team member (S.B.), needed to check their smartphones’ compatibility and perform the setup elsewhere in the hospital, on any chair or bench that was free. This was not ideal for the children or their parents, because they had travelled several hours to attend the outpatients clinic. Parent 2 described that they had a list of errands to attend to “while in the big city”, and Parent 1 “need[ed] to get back [home] before dark”. This resulted in several participants postponing the setup to a later date and the intervention’s instructions being provided over the phone. All these interferences had a detrimental effect on the pre-education and practice which was needed to support children with useing the intervention.

### 3.2. Theme 2: Personal Phone Restrictions

During the setup phase of the intervention, we soon identified low smartphone ownership rates by children (0%, 0/5). This meant that all children needed to synchronise the smartwatch to their parent’s smartphone. Two main sub-themes emerged: parents’ digital health literacy, and personal phone inconvenience.

#### 3.2.1. Parents Digital Health Literacy

All parents were required to download the required apps onto their smartphone to enable synchronisation of the smartwatch to the chronic disease web-based platform. To achieve this, parents were required to log into an App Store, however a common set of behaviours soon emerged.

“I have no idea of my App Store password, I even tried my wife’s phone” (Parent 1)

“I’ve never used the App Store” (Parent 3)

It soon became clear that parents were not regular digital app consumers. Most parents found this process to be frustrating and had trouble downloading the required apps. One parent in particular described the App Store setup process as “devastating”, due to the fact they were asked to upload their credit card details. They were unaware that this section of the App Store signup was not compulsory, and as a result, dropped out of the study.

#### 3.2.2. Parental Phone Inconvenience

Children’s engagement with the intervention was limited because of the smartwatch being synchronised to their parent’s phone. For example, one father left for work at 7 a.m. and did not return home until 6.30 p.m. For this younger child, aged 10 years, “This was close to bed” (Child 2). Another child reported not using the app, because “I am at my dad’s [house] this weekend” (Child 3). While visiting a separated or divorced parent, this child could not use the app on their other parent’s phone. This parental phone inconvenience resulted in a further two children dropping out of the study.

### 3.3. Theme 3: Elimination of the Parent’s Phone

During a research team meeting, while reviewing the intention of this study and child and parent feedback, it was identified that relying on parents’ phones to deliver the intervention was having a major impact on the study. Parental digital health literacy (app-store setup, 20%, one-fifth) and parental phone inconvenience (40%, two-fifths) had contributed to the drop out of three participants (60%, 3/5). Consequently, a decision was made to stop using the parent’s phone.

Instead, to promote active participation in the study and support the intervention’s development, the remaining two children were provided with an already setup smartphone by mail, which was loaded with AUD $30 of prepaid data to support engagement (ethics approved). This transferred the focus of setup, to education and practice using the watch and phone. The researchers’ (S.B.) contact details were also entered into the phone, and they were encouraged to ring or text if they were experiencing any problems with the setup. Without prompting, text messages were sent by the two remaining participants, updating the research team of their progress, after receiving the phone.

“hey, its XXXX, watch is going very well so far” (Child 4)

“all good ☺” (Child 5).

## 4. Discussion

The main purpose of this exploratory study was to investigate the challenges experienced during the setup phase of a smartwatch intervention, designed to support children living with a chronic health condition. This was achieved by examining the digital landscape of an Australian public hospital subspecialty outpatient’s clinic within a specialised tertiary referral paediatric hospital in a regional city of NSW.

Several barriers were identified that digital innovators may face, deploying a digital health intervention using smartwatch technologies. These barriers resulted in the formation of three themes: infrastructure limitations, personal phone restrictions, and the elimination of the parents’ phone.

First, infrastructure limitations resulted because of inconsistent interoperability between the smartwatch and parents smartphones. This exploratory study utilised a hybrid model to deliver the digital health intervention, which means it was reliant on healthcare services and public resources. Therefore, it was dependant on connecting the smartwatch to a personally owned smartphone, utilising an Android operating system (OS). OS is the world’s leading mobile operating system, with a market share of 70.7% [41]. Before the study, all smartwatches were pretested using an OS smartphone. This phone cost AUD $300.

However, it soon became clear on the first day of the study; children did not own a smartphone to support the smartwatch, and parents more economical OS smartphones were incompatible with smartwatch technology. The exclusion of lower cost smartphones was not an area we considered in the exclusion criteria, especially considering that we were not directly testing the watch or phone, but rather the intervention. Notably, the Alliance for Affordable Internet [AFAI] reporting on the cost and features of smartphones across 187 countries revealed that lower-cost smartphones often represent earlier models and support only basic applications [42]. If the biometric data collected from a smartwatch is to be considered as a supportive health intervention, the expectations of using personal phones may not match ownership. In the US, for example, 30% of people pay less than USD $300 for their phone [43]. Excluding those who do not have access to an appropriate device or adequate internet could further marginalise under-resourced populations, creating a digital divide [44,45,46]. For children living with a chronic health condition, we need to consider the financial impact this can have on a family’s income [47]. Therefore, if digital healthcare aims to close the gap on the current health disparities that exist across the globe in healthcare delivery and improve quality of life [46], it is very important to ensure equity of access, especially for children.

From a public healthcare perspective, another important barrier to acknowledge is the hospital’s digital landscape. If digital health technology is to be utilised in clinical practice, the infrastructure within a healthcare service needs to ensure no internet black spots. In this study, the location of the outpatient’s clinic impaired internet connectivity, hindering the setup phase and limiting access and training of the intervention. A recent scoping review identified that, if technical factors interfere with a digital intervention’s workflow, they can directly impact the implementation and acceptance of the intervention [24]. Therefore, it is no wonder that digital health researchers often opt to supply the equipment needed, such as webcams, software packages, and loan computers, with prepaid wi-fi to conduct their studies [24]. Acknowledging the need for these resources is important to enable digital researchers to plan ahead and to move the development of digital healthcare forward [24]. For this study to progress, a smartwatch, pre-configured smartphone, and cellular data (AUD $30, EUR €18) needed to be supplied to the children, to support training and engagement with the intervention for children.

It is also imperative that each country ensures they have the correct support and infrastructure in place to become digitally prepared [46]. An emphasis needs to be placed on the importance of achieving a fully interoperable healthcare ecosystem to improve the exchange of health data and provide patients with the best possible care [48]. For example, in Australia, since 2022, NSW Health has been using the eHealth NSW’s Health Wide Area Network (HWAN) to ensure infrastructure consistency across their health services [49]. They are also offering a state-wide patient and guest Wi-Fi service to improve the inpatient experience [49]. However, for this study, connecting to this service was not possible in the Outpatients Clinic due to the hospital’s design reducing Wi-Fi signals. Importantly, this identifies a need for further improvement to support the delivery of digital health in all areas of healthcare and to ensure that healthcare management, policy-makers and other stakeholders are aware of these current barriers and take action [48].

Equally important is to address the personal phone restrictions, identified in theme 2 in this study. Poor levels of digital literacy identified by parents also hindered setup, which removed the opportunity for face-to-face, hands-on training. Other studies have shown that training and education are important to improve literacy [50,51] and facilitate meaningful participation with digital health [50]. This is imperative, because if patients do not have access to the required digital technology, do not understand digital health capabilities, or are unaware of the available digital support, this can intensify health inequality [51]. Therefore, there is a shared responsibility for healthcare professionals, healthcare organisations, governments, and private stakeholders to combat this digital divide by forming potential solutions that facilitate or subsidise digital health support [19,50].

Unfortunately, a more difficult problem to overcome in this study was the need to use the parent’s phone, to deliver the intervention. Since none of the children owned their own phone, this created a significant barrier, impairing notification delivery to the smartwatch and engagement with the app, when children were not in close proximity their parent’s phone. Statistics suggest most Australian’s own a mobile phone, but this is not the reality for children. In fact, 54% of children (aged 6–13 years) do not own or have access to a phone [52]. Phone ownership typically begins at aged 13.1 years, when parents feel safe in allowing their child to share their information with an online service (aged 13.3 years) [53]. Ownership rates then steadily increase from 33% (aged 6–13 years) [52] to 81% between 14 to17 years [54]. Interestingly, another study on self-managing blood glucose levels (BGL) also reported problems when they used the parent’s phone [55]. Parents were able to view their child’s BGL results on their phone, which eased their concerns. However, for the child, this increased conflict, because parental reminders increased if BGL measurements were missed, and as a result of this parental surveillance, some children developed oppositional behaviour, undermining further BGL assessments and their BGL control [55]. This highlighted several concerns associated with utilising a parent’s phone to support children. Indeed, this is an area that needs more resources and rigorous research.

### Limitations

There are several limitations associated with this exploratory study. First, this study only used a small sample size of five children and five parents, which can have an impact on internal validity. However, a sample size of five is commonly used when cyclically testing different stages of a digital intervention’s development. In fact, five participants can detect 80% of the problems [56], because the most severe problems are identified by the first few users [57]. These problems can then be addressed, and the intervention retested, using different participants [56]. The World Health Organization (WHO) endorses this incremental approach of testing for children to support early planning and prototype development [58]. The recruitment of a large number of participants was also restricted due to the COVID-19 pandemic. All non-essential research was paused in the hospital setting when this study was to commence, reducing the timeframe allocated to this research.

Second, this study was conducted in an Outpatients Clinic in a specialised tertiary referral paediatric hospital in a regional city, which hinders its transferability. This is a common limitation of qualitative research, because it places the study findings within a given context. However, the Australian Communications and Media Authority reports little difference in the ownership of smart devices by Australians in metropolitan (85%) and regional (81%) areas [59], suggesting that the device barriers identified in this study may be widespread. Large-scale testing at the time of this study was not feasible, because potential study funds had been rightfully diverted to support the COVID-19 pandemic. However, to verify these rates, specifically for children living with a chronic health condition, a survey will now be conducted across all tertiary children’s hospitals in Australia. This would allow for the further exploration of Australia’s digital landscape and the practicality of utilising a hybrid model (personally owned smartphones and smartwatches) targeting children. This approach would help on a wider scale, to identify the resources needed to utilise the capabilities of smartwatch technology and digital health for children. Raising awareness of these barriers hindering information exchange is important so that healthcare management and policy makers can take action [50].

Third, there were several limitations with the methodology used, which was document analysis. Documents might not correctly represent all the events that occurred [60]. Therefore, all the documentation acquired over the study period from the participants, parents, and the research team were utilised to gain a range of perspectives. However, the retrieval of data from the documents can also be subjective. To reduce the risk, using READ’s four steps of data analysis supported a clear data trail and ensured transparency and rigour at all stages of the analysis [40]. Final themes were also internally member checked to ensure the trustworthiness of the results. To further lay the foundations for a digital health intervention for children, the WHO recommends that the next stage of planning after a landscape analysis should be a needs assessment [58]. This assessment will help identify the health parameters a digital intervention must target, thereby informing the collection of health data to support health outcomes for children living with a chronic health condition. However, both the landscape analysis and needs assessment must be continually re-addressed due to the rapidly evolving digital landscape and changing needs of users [58].

Finally, providing children with an internet-enabled device to engage with a digital health intervention has privacy and security risks. However, to mitigate these risks and promote online safety, strict content and privacy restrictions were placed on screen time [61], and all the used smartphones’ phone numbers were registered in the Australian Communications and Media Authority’s Do Not Call Register to prevent unsolicited telemarketing [61]. Further, to ensure effective cyber security, secure network protection was gained by using an Australian server to deliver, process, and store all study data. Importantly, this area requires ongoing attention from future digital health innovators to ensure they remain up to date with the current policies and services that are available. Many governments are currently reviewing existing laws and regulations to streamline processes and ensure better data safety for children [62,63].

## 5. Conclusions

The findings of this exploratory study identified several challenges for digital researchers to consider if smartwatch technology aims to support children living with a chronic health condition. First, if an intervention is reliant on using a hybrid model to deliver the digital health intervention, it is crucial to pre-examine the digital landscape required to support the intervention. This study identified that lower-cost smartphones were incompatible with smartwatch technology and prevented connection, and the Outpatients Clinic was in an internet black spot, constraining setup and training. Second, using a parent’s phone hindered the setup due to low digital literacy; and access to the intervention when the child was not with their parent. Therefore, the solution to these barriers was to stop using the parent’s phone. Instead, children were supplied with the digital support needed to participate (a smartphone, pre-installed apps, and phone data). Overall, we identified several barriers that could hinder the use of digital health in clinical practice. To ensure paediatric preparedness for newly emerging smart technologies, further research is needed, to gain a more detailed understanding of the resources required to support digital healthcare for children living with a chronic health condition.

## Data Availability

The data presented in this study is available on request from the corresponding author. The data are not publicly available due to the research being conducted on children.
